# Exploring the Complexity of the HIV-1 Fitness Landscape

**DOI:** 10.1371/journal.pgen.1002551

**Published:** 2012-03-08

**Authors:** Roger D. Kouyos, Gabriel E. Leventhal, Trevor Hinkley, Mojgan Haddad, Jeannette M. Whitcomb, Christos J. Petropoulos, Sebastian Bonhoeffer

**Affiliations:** 1 ETH Zürich, Institute of Integrative Biology, Zürich, Switzerland; 2 Department of Ecology and Evolutionary Biology, Princeton University, Princeton, New Jersey, United States of America; 3 Monogram Biosciences, South San Francisco, California, United States of America; University of Arizona, United States of America

## Abstract

Although fitness landscapes are central to evolutionary theory, so far no biologically realistic examples for large-scale fitness landscapes have been described. Most currently available biological examples are restricted to very few loci or alleles and therefore do not capture the high dimensionality characteristic of real fitness landscapes. Here we analyze large-scale fitness landscapes that are based on predictive models for in vitro replicative fitness of HIV-1. We find that these landscapes are characterized by large correlation lengths, considerable neutrality, and high ruggedness and that these properties depend only weakly on whether fitness is measured in the absence or presence of different antiretrovirals. Accordingly, adaptive processes on these landscapes depend sensitively on the initial conditions. While the relative extent to which mutations affect fitness on their own (main effects) or in combination with other mutations (epistasis) is a strong determinant of these properties, the fitness landscape of HIV-1 is considerably less rugged, less neutral, and more correlated than expected from the distribution of main effects and epistatic interactions alone. Overall this study confirms theoretical conjectures about the complexity of biological fitness landscapes and the importance of the high dimensionality of the genetic space in which adaptation takes place.

## Author Summary

Evolutionary adaptation can be understood as populations moving uphill on landscapes, in which height corresponds to evolutionary fitness. Although such fitness landscapes are central to evolutionary theory, there is currently a lack of biologically realistic examples. Here we analyze large-scale fitness landscapes derived from in vitro fitness measurements of HIV-1. We find that these landscapes are very rugged and that, accordingly, adaptive processes on these landscapes depend sensitively on the initial conditions. Moreover, the landscapes contain large networks along which fitness changes only minimally. While the relative extent to which mutations affect fitness on their own or in combination with other mutations is a strong determinant of these properties, the fitness landscape of HIV-1 is considerably less rugged than expected from the individual and pair-wise effects of mutations. Overall this study confirms theoretical conjectures about the complexity of biological fitness landscapes and the importance of the high dimensionality of the genetic space in which adaptation takes place.

## Introduction

The fitness landscape is one of the central concepts in evolutionary biology. Ever since Sewall Wright [1], it has been used to study and conceptualize the process of long-term evolutionary adaptation. Fundamentally, knowledge of fitness landscapes is required to translate microevolutionary adaptation (i.e. changes in gene frequencies) into macroevolutionary change (i.e. speciation events and large-scale phenotypic modifications). One of the major limitations of the concept of fitness landscapes, however, is the near complete lack of knowledge of any large-scale and biologically realistic fitness landscapes. Most of the landscapes currently available are restricted to very few loci or alleles [2], [3], [4], [5], [6], [7], [8]. Due to their limitation in size, these landscapes do not allow the study of properties that might arise from the high dimensionality that is characteristic for real fitness landscapes. Current examples for large-scale landscapes are based on RNA secondary structure [9] or enzymatic activity of RNA [10]. However, the relation of RNA structure to fitness is unclear and the relation between enzymatic activity and fitness is often highly non-linear [11].

The centrality of the concept of fitness landscapes for evolutionary biology, combined with the absence of good biological examples has necessitated the study of theoretically conceived and idealized fitness landscapes, often tailored to the particular question studied. The so-called NK landscapes are an example for a broad class of theoretical fitness landscapes [12], which have tunable ruggedness ranging from smooth, single-peaked Mount-Fujiyama-like landscapes to maximally rugged uncorrelated landscapes, in which the fitness of each sequence is independent of the fitness of its neighbors. These NK landscapes have been used, among other things, to study properties of landscapes arising from high dimensionality [13]. Landscapes based on neutral networks [14], [15] reconcile Kimura's neutral theory [16] with natural selection, and have been used to explain phenomena such as punctuated equilibria observed for example in the evolution of the antigenic profile of influenza [17]. The related holey landscapes, which consist of a network of high-fitness genotypes with embedded fitness-holes, have been very influential as models of speciation [18]. All these examples have been tremendously valuable in studying processes of evolutionary adaptation, but are purely conceptual and it is unclear to what extent they reflect properties of real fitness landscapes.

Recent progress in high throughput data generation now allows measuring both fitness and genotype for a large number of mutants [19], [20], [21]. Combining such data sets with appropriate computational methods enables for the first time the reconstruction of large-scale and biologically realistic fitness landscapes. Here we analyze fitness landscapes that are based on predictive models for fitness of HIV in an in vitro replication assay [21]. These models predict fitness based on estimated effects of individual mutations (main effects) and of pair-wise combinations of mutations (epistasis) and can thus be considered as a quadratic approximation to the real HIV fitness landscape.

## Results/Discussion

The fitness landscapes analyzed here are based on statistical models that are based on extensive measurements of *in vitro* replicative fitness. These models allow to predict the fitness of HIV from amino acid sequences (see Materials and Methods and ref. [21]). The entire landscape consists of approximately 2^1800^≅10^600^ fitness values. Clearly, it is impossible to generate all these values despite the fact that the predictive model would allow in principle to compute the fitness for any sequence. Therefore, we describe the properties of the fitness landscapes by using summary statistics based on different types of random or directed walks on these landscapes. Specifically we use such walks to compute three measures that characterize different properties of the landscapes: ruggedness, correlation length and neutrality (see Materials and Methods).

Ruggedness refers to the number of local fitness optima; i.e. genotypes whose fitness exceeds that of every one of its neighbors. We determine ruggedness as the number of different local optima reached by adaptive walks that climb the fitness landscape by means of steepest ascent from random positions on the landscape. These adaptive walks always move to that neighboring sequence, which has the highest fitness of all the neighboring sequences. Local optima act as attractors for such steepest-ascent walks: if a walk is started within the “attraction domain” of the optimum, the walk will converge to this optimum. Depending on the structure of the landscape, such walks need not end up in the same optima, even if they are started from similar initial conditions. Conversely, walks that end up in the same optima need not originate from similar areas of the fitness landscape. We use such simple hill-climbing walks here as tools to analyze structural properties of the underlying fitness landscape such as ruggedness or the attraction domain of the local optima. To characterize the process of adaptation of populations evolving on these fitness landscapes such hill-climbing walks have limited validity and may overly simplify more complex aspects of evolution. The correlation length quantifies to what extent proximity in sequence space translates into similarity in fitness. To measure correlation length, we perform random walks, which start at a random genotype in the landscape and then randomly move in each step to neighboring genotypes. Recording the fitness values along such a random walk we then determine correlation length as the characteristic distance over which the autocorrelation of fitness decays. Neutrality measures to what extent populations can move on the landscape without changing their fitness. To measure neutrality, we perform quasi-neutral walks, where random steps to neighboring genotypes are only accepted if they do not change fitness by more than a defined small threshold value. We determine neutrality as the maximal distance from the starting genotype that is attained by such a neutral walk.

We first explore these measures for a reference landscape (RL), which is based on the model that best predicts replicative capacity in the drug-free environment (see Materials and Methods and ref. [21]). We then examine different variations of the RL (see Materials and Methods) in order to explore how these measures depend on the features of the underlying landscape. These variations include landscapes based on fitness measured in the presence of different antiretroviral drugs; landscapes, in which the strength of epistasis is reduced; and landscapes in which the coefficients determining main effects and pair-wise epistasis in the RL are randomized (see below).

**Figure 1:**
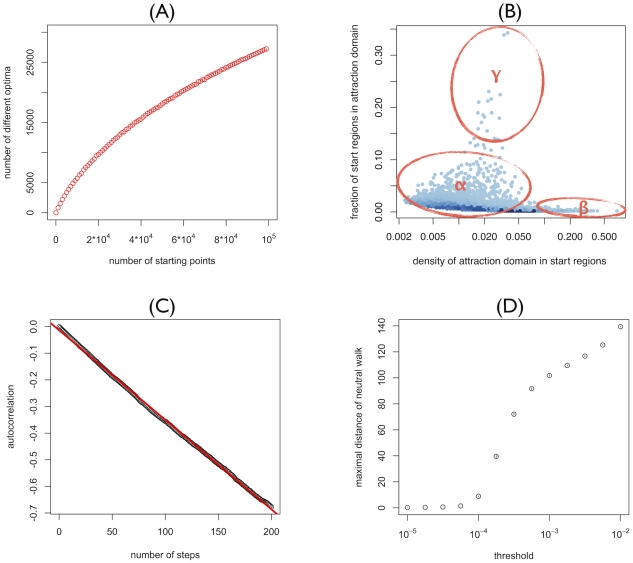
Properties of the reference landscape. (A) Number of different optima attained from steepest-ascent hill-climbing walks starting from random genotypes plotted as a function of the number of starting genotypes. (B) Distribution of attraction domains of steepest-ascent hill-climbing walks: Starting genotypes are chosen in the neighborhoods of 500 randomly chosen reference genotypes. Of each reference genotype, 100 random single, double, triple, fourfold, and fivefold mutants are considered as starting genotypes. Each dot corresponds to a local optimum. Coordinates indicate from how many unique neighborhoods (y-axis) and from what fraction of starting-genotypes in these neighborhoods the optimum is reached (x axis). Thus the y- and x-axis correspond to the global and local density of the attraction domain respectively. (C) Autocorrelation of log-fitness along random walks as a function of the number of steps. The red line corresponds to the linear least square fit of the autocorrelation and the correlation length is given by −1/(slope of the line). (D) Range explored by quasi-neutral walks for different discrete values of the maximal fitness-effect *ε* which quasi-neutral mutations are allowed to have. Points correspond to the mean over 10^5^ walks of length 1000. 95%-confidence-intervals of the mean (inferred through 1000 bootstrap samples) are smaller than point size.

The RL is characterized by a large number of optima, a large correlation length and considerable neutrality (Figure 1). For an increasing number of starting points we find an increasing number of optima, with only a weak saturation of the increase up to 10^5^ different starting points. For the 10^5^ starting points tested in Figure 1, on average every fourth one leads to a different optimum. By contrast, in a completely smooth landscape such walks would always converge to the same optimum. The large number of optima indicate (Figure 1A) a high degree of ruggedness and hence a multitude of basins of attraction (i.e. the set of starting points from which adaptive walks end up in a given optimum). Moreover, the starting genotype of an adaptive walk has a strong influence on the long-term evolutionary trajectory and neighboring starting points can lead to completely different trajectories (Figure 1B). The basins of attraction of the different optima differ greatly in structure. Some optima have an attraction domain that is confined to a small region of sequence space and is sparsely distributed within this region (type α in Figure 1B). Other optima have an attraction domain, which also covers a small region of sequence space, but is densely distributed in that region (type β). Finally, optima of the last type have an attraction domain that spans a large part of sequence space but is only sparsely distributed (type γ). The long correlation length of random walks on the RL (Figure 1C) indicate that almost all loci characterizing a genotype (here, genotypes consist of 404 loci, see Materials and Methods) have to be mutated until the memory of the initial fitness value is lost.

In the landscapes considered here, mutations may have extremely small effects, but they are never completely neutral. To define a sensible concept of neutrality we therefore need to define a threshold for the maximal fitness effect that a mutation is allowed to have to be considered neutral. The exploration range of the resulting quasi-neutral walks strongly depends on the magnitude of this threshold (see Figure 1D). If this threshold is 10^−4^ or lower, the exploration range is very small with a maximal distance of 5–10 mutations. For thresholds of 10^−3^ or higher, on the other hand, neutral walks can reach considerable distances of 100 mutations or more. Thus, although there are no fully neutral mutations in the RL, the landscape is characterized by large networks over which fitness changes only minimally.

**Figure 2:**
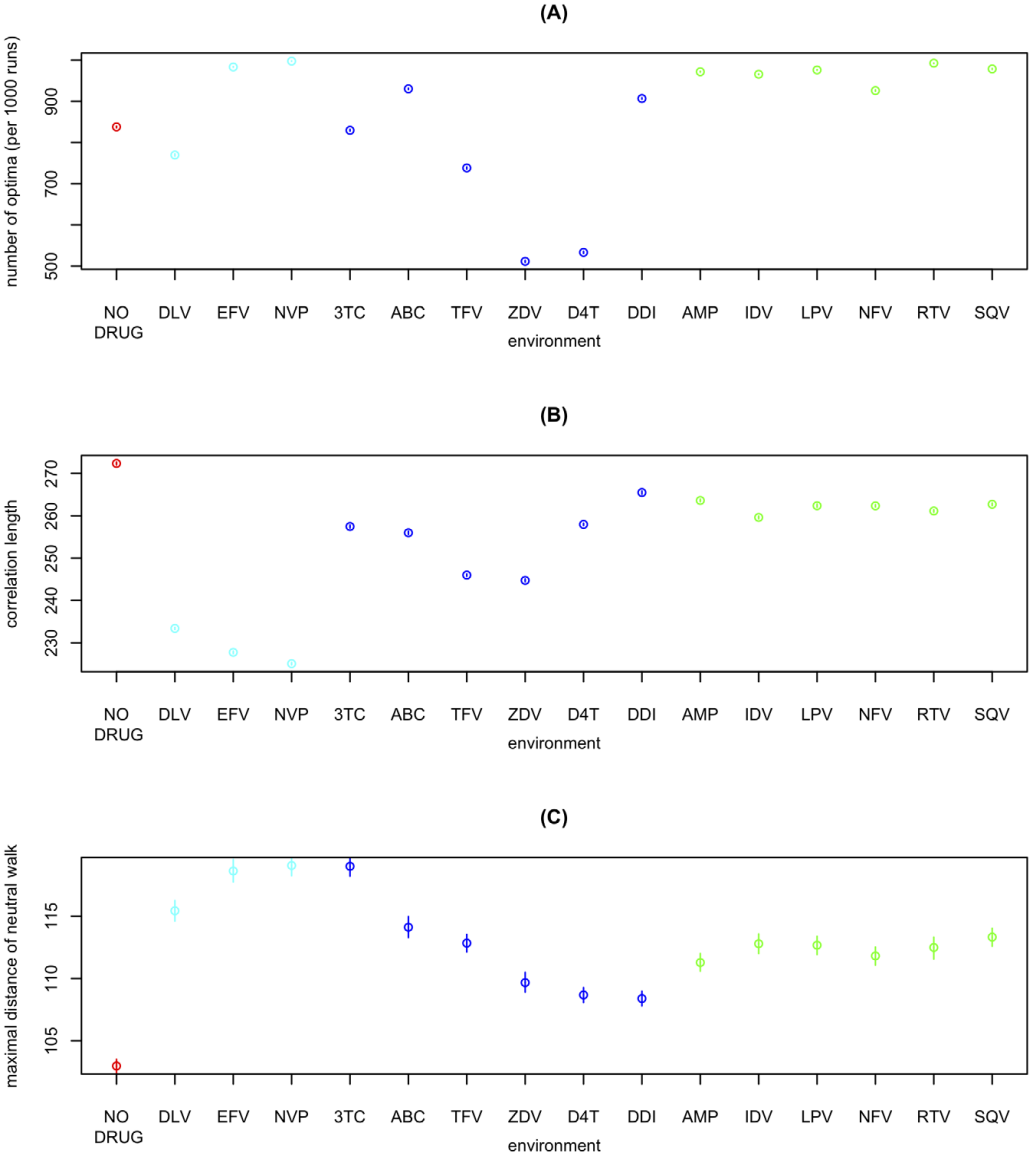
Fitness landscapes across different environments characterized by the absence of drugs or by the presence of a single antiretroviral. (A) Ruggedness (i.e. number of different optima reached from 1000 steepest-ascent hill-climbing walks) for the no-drug and 15 single-drug environments. X-axis labels indicate the antiretroviral drug characterizing each environment (see Materials and Methods) and color indicates drug-class (red: no drug; cyan: non-nucleoside reverse transcriptase inhibitor; blue: nucleoside analog reverse transcriptase inhibitor; green: protease inhibitor). Each point corresponds to the mean over 100 such measures of ruggedness. 95%-confidence-intervals of the mean (inferred through 1000 bootstrap samples) are smaller than point size. (B) Correlation-length of log-fitness on random walks. Correlation-length is inferred from 10^4^ random walks of length 50 starting from random initial conditions. Points correspond to the mean over 100 such measurements of correlation length. 95%-confidence-intervals of the mean (inferred through 1000 bootstrap samples) are smaller than point size. (C) Range explored by quasi-neutral walks (threshold ε = 0.001) for different environments. Points correspond to the mean over 10^5^ walks of length 1000. Error-bars correspond to the 95% confidence-interval of the mean, inferred through 1000 bootstrap samples.

Comparing the RL to the corresponding best-fit landscapes for 15 different environments each characterized by the presence of a different antiretroviral drug (see Materials and Methods), we find that ruggedness, correlation length, and neutrality are of similar magnitude in all these environments (Figure 2). However, the no-drug environment exhibits less neutrality and longer correlation length than all environments with antiretrovirals present. Interestingly, there are also consistent differences between drug-classes. Figure 2 shows results for the 3 main classes of antiretroviral drugs: protease inhibitors (green), nucleoside reverse transcriptase inhibitors (blue), and non-nucleoside reverse transcriptase inhibitors (cyan). For instance, the landscape is particularly rugged in the presence of protease inhibitors. Interestingly, protease inhibitors are also known to have the most complex resistance profiles [22], [23], [24], in the sense that resistance against them is mediated by a large number of interacting mutations.

To assess the impact of the strength of epitasis relative to that of main effects, we consider alternative landscapes in which fitness interactions between mutations are weaker. We chose the RL as a reference because it has the highest predictive power (see [21] and Figure S1). However, this landscape might overestimate the role of epistasis for statistical reasons (see Materials and Methods). A landscape based on a more conservative estimate of the role of epistasis can be obtained by fitting a hierarchical model that first estimates the effects of individual mutations (“main effects”) and then uses pair-wise interactions between mutations (“epistasis”) to explain the remaining variance in the biological data (see Materials and Methods). This hierarchical landscape (HL) has a predictive power almost equal to that of the RL (see Materials and Methods). As both the RL and the HL represent equally valid approximations of the true biological fitness landscape, the true magnitude of epistasis will presumably lie between these two extremes. To further explore the role of epistasis we can reduce its magnitude beyond the realistic range by reducing all epistatic interactions in the HL by a factor *ε* (0≤*ε*≤1). The predictive power of the corresponding landscapes (HL_ε_) decreases for small *ε*, but even for *ε* = 0 the predictive power is only reduced by 13% (see Materials and Methods). This continuum of the HL_ε_ landscapes allows us to study the effect of the relative strength of epistasis on ruggedness, correlation length and neutrality.

**Figure 3:**
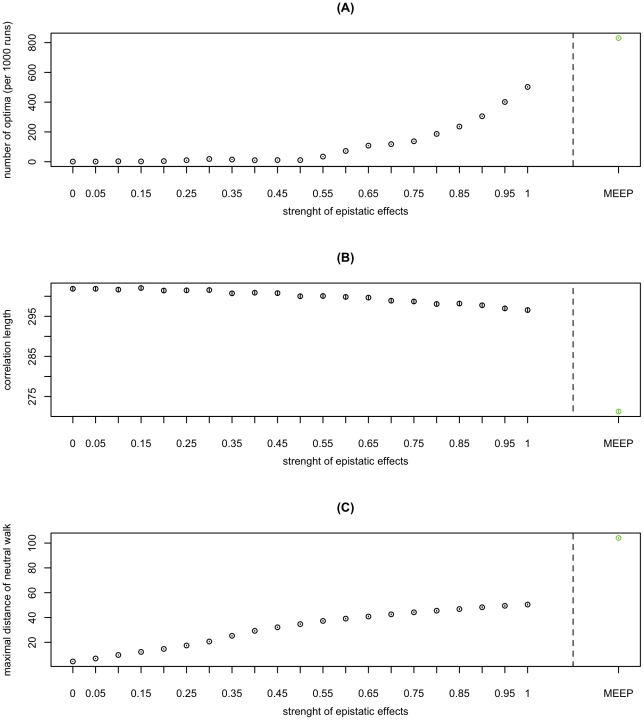
The impact of epistasis on the structure of fitness landscapes. Ruggedness (A), correlation length (B), and neutrality (C) as a function of the magnitude of epistasis in HL_ε_. For all panels, the 95% confidence interval of the mean (inferred through 1000 bootstrap samples) is smaller than the size of the data point symbol.

We find that ruggedness and neutrality consistently increase with the magnitude of epistatic effects (Figure 3). With increasing epistasis, the HL_ε_ gradually shift from a single-peaked smooth landscape without neutral networks (*ε* = 0) to a very rugged landscape with large quasi-neutral networks (*ε* = 1). In contrast to the other two measures, correlation length in the HL_ε_ depends only weakly on epistasis. All three measures continue to exhibit the same type of dependence on epistasis when switching from the HL to the more epistatic RL (Figure 3). Taken together, these results indicate that the relative strength of pair-wise epistasis is a major determinant of the structure of fitness landscapes.

An intuition for the impact of the strength of epistasis on ruggedness can be obtained as follows: If main effects dominate, a given mutation is always either beneficial or deleterious, independent of its background. However, if epistatic interactions dominate, a change in the genetic background can turn a beneficial mutation into deleterious one and vice versa. Thus the landscape only has one peak if main effects dominate, but may have multiple peaks if epistatic effects dominate. Note that epistasis need not necessarily increase ruggedness. For example, this would not be the case if most epistatic interactions were of the same sign (as has often been assumed [25], [26]). Thus the increase in ruggedness with epistasis is a particular feature of the landscapes studied here. The fact that neutrality increases with epistasis might seem contradictory at first, given that epistasis contributes to the selective effects of mutations. One should note, however, that non-trivial neutrality (i.e. a mutation being neutral in some genetic backgrounds but not in others) requires epistasis by definition. This type of non-trivial neutrality is responsible for the observed increase in neutrality with increasing epistasis. In contrast to the trivial type of neutrality that would be due to synonymous mutations, the neutrality observed here is exclusively due to the cancelling out of selective effects. Finally, the correlation length of a random walk decreases with the strength of epistatic effects for the following reason: In the case of independent loci, correlation is lost if on average every locus has been mutated. If loci interact epistatically, then a mutation at one locus affects the fitness-contribution of the mutations at other loci as well and hence the number of changes required to loose correlation decreases. Given these interpretations, our results (that ruggedness and correlation length are high) suggest that epistasis is strong enough to increase ruggedness, but too weak to strongly affect correlation length. Interestingly Fontana et al. [9], [27] found that landscapes in which fitness is predicted by RNA secondary structure combine neutrality and ruggedness similarly to the landscapes described here. However, these RNA-derived landscapes exhibit short correlation lengths, in contrast to our HIV landscapes. As correlation length decreases with the strength of epistatic interaction (see Figure 3), one reason for this difference might be that the epistatic or pair-wise interactions are much stronger in RNA landscapes than in the HIV landscapes analyzed here.

**Figure 4:**
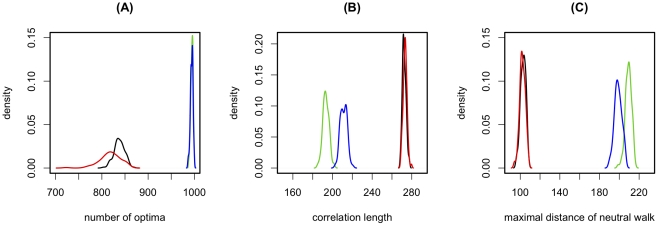
The impact of the correlation structure of epistasis on the fitness landscape. Distribution of ruggedness (A), correlation length (B), and neutrality (C), for different randomizations of the reference landscape. The following randomization schemes are used: In scheme 1 we draw main effects randomly with replacement from the distribution of main effects underlying the RL, whereas epistatic effects are kept as they are in the RL. This destroys any correlation between epistasis and main effects. In scheme 2 we additionally shuffle the non-zero epistasis values. This retains the information of which loci interact epistatically, but shuffles the value of any such interaction. Finally, in scheme 3, we fully shuffle all epistasis and main effect values, and thus destroy all correlations between effects. Each measure is inferred for 100 randomizations of each randomization type and the interpolation of the resulting distribution is plotted. : No randomization (i.e. the 100 realizations are done on the same landscape; black), scheme 1 (red), scheme 2 (blue), scheme 3 (green). For the latter two cases it should be noted that main effects and epistatic effects are shuffled separately, i.e. main effects remain main effects and epistatic effects remain epistatic effects.

The strong impact of the relative strength of main effects and epistasis raises the question whether the properties of fitness landscapes also depend on the detailed correlation structure between different epistatic effects and main effects or whether they are only determined by the distributions of these effects. In order to address this question, we use three different schemes to randomize the main and epistatic effects underlying the RL (Figure 4) and then measure ruggedness, neutrality and correlation length for the different types of randomized landscapes. We find that, despite its large number of peaks, the RL is still considerably smoother (smaller number of peaks) than the randomized landscapes (see Figure 4A). Furthermore, the RL is also less neutral and more correlated (see Figure 4C and 4B). This implies that the fitness landscape of HIV is considerably less rugged, less neutral, and more correlated than expected from the distribution of main effects and epistatic interactions alone. Moreover, it suggests that, although the overall strength of epistasis is an important factor, knowledge of the distributions of main effects and epistatic interactions does not fully characterize fitness landscapes in general, because the correlational structure between epistatic effects plays an essential role in determining the properties of the landscapes.

It should be noted that the structure of the fitness landscapes discussed here might be affected by selection biases in the data used for the development the fitness-prediction model. The viral isolates have been obtained from HIV-infected individuals and therefore the mutations found in these isolates do not represent a random sample of all possible mutations. On the one hand, because all isolates harbour replication competent viruses, the sample is biased against lethal or highly deleterious mutations. On the other hand, most viral isolates carry drug resistance mutations. These resistance mutations are beneficial in the presence, but typically detrimental in absence of drugs. Hence, in the drug free environment (or in an environment containing drugs to which a give mutation does not confer resistance) the isolates may be enriched in deleterious mutations. In any event, the mutations found in the isolates represent the standing variation of mutations that are present on the level of the host population. Clearly, however, it is likely that the complete fitness landscape of HIV does contain much more fitness-holes/troughs than the landscapes described here, because of the observation bias against lethal mutants.

Comparing the fitness landscape of HIV with various theoretical landscapes that have been used to study evolutionary processes [12], [14], [15], [18] shows that these classical landscapes can capture certain aspects of a real landscape while failing to describe others. For example, the fitness landscape of HIV resembles uncorrelated landscapes with regard to the high ruggedness. However, unlike uncorrelated landscapes, it is characterized by considerable neutrality and large correlation length. In these respects, the HIV fitness landscape is closer to neutral landscapes (such as holey landscapes) or single-peaked Mount-Fujiyama-like landscapes. Finally, the structure of the attraction domains—in particular the existence of attraction domains which are at the same time very sparsely and widely distributed—strongly contrasts the situation in low dimensional spaces. Overall, these results highlight the complexity and the high dimensionality that need to be taken into account to describe adaptive processes in real biological systems.

## Materials and Methods

### Fitness Landscapes

The fitness-landscapes analyzed here are based on models that predict the fitness of HIV from amino acid sequences. Fitness is measured as the reproductive capacity (RC) of HIV-derived amplicons (representing all of Protease (PR) and most of Reverse Transcriptase (RT)) inserted into a constant backbone of a resistance test vector. The models are then trained to predict this fitness from the amino-acid sequence of the amplicons. Although the fitness, which is predicted by these models, is an in-vitro RC, we could show in [28] that this predicted RC is significantly correlated to HIV virus load in vivo. Details on the experimental measurement of the RC values and on inferring the predictor have been published in [29] and [21]. Here, we briefly reiterate the principles of the models fitted to the data.

In essence, the predictor is based on fitting the data consisting of amino acid sequences *(s)*, coded here as a binary string, and the corresponding RC values (*w*) with the following model
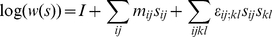
(M1)For the purpose of this paper, *s_ij_* denotes the presence (*s_ij_* = 1) or absence (*s_ij_* = 0) of allele j at position *i*. (Although the present work is restricted to this simple binary case, a more general definition is used in the data fitting procedure [21]: If an ambiguity in the population sequencing is consistent with several amino acids at a given position, then *s_ij_* denotes the probability of allele j at position i.) Thus *s* is a valid sequence only if for all positions *i*, 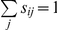. In total, there are 1859 alleles at 404 positions. The vast majority (1848/1859) of these alleles are amino acids (thus not all possible amino acids at the 404 positions are allowed); only 11 alleles correspond to either insertions or deletions. The model parameters *I*, *s_ij_* and *ε_ij;kl_* can be interpreted as intercept, main effects (effects of individual alleles on their own), and epistatic effects (effects of an allele at one locus in combination with an allele at another locus). As the number of parameters exceeds the number of data-points, the model *M1* has been fitted to the data on the basis of a machine learning approach (generalized kernel ridge regression). With this approach over-fitting is no concern because the sub-dataset on which the predictor is evaluated is independent from the sub-dataset from which the predictor is inferred (see [21]).

Note that equation (M1) can also be written as a second order cluster expansion [30] of the log-fitness
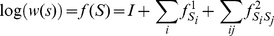
(M2)where *S_j_* denotes the allele at position *j* of the amino-acid sequence, 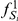 the impact on log-fitness of allele *S_i_* at position *i*, and 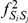 denotes the combined impact of allele *S_i_* at position *i* and *S_j_* at position *j*. The first-order effects 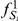 in equation (M2) correspond to the main-effects in equation (M1) and the second order effects 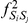 to the epistatic effects in equation (M1). For instance if *i* is a bi-allelic locus with alleles *S_i_/S_i_'* and *k/k'* denote the position corresponding to those alleles in the binary representation used above, then 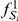 = *m_k_* and 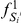 = *m_k'_*.

The different landscapes are all based on model M1, but differ with respect to the relative weight that is given to epistasis and main effects:

- The reference landscape RL is obtained by fitting the full model M1 to the data. Thus main effects and epistatic effects are fitted simultaneously. As main and epistatic effects are given the same weight in model fitting, while epistatic effects greatly outnumber main effects, this approach will explain the variance in RCs using mainly epistasis. Therefore this approach tends to overestimate the role of epistasis relative to that of main effects.
- The hierarchic landscape (HL) avoids this overestimation of epistatic effects by first fitting model M1 only with the main effects (i.e. the *ε_ij;kl_* in M1 are set to 0) and then fitting the residuals of the first fit only with the epistatic effects (i.e. *m_ij_* in M1 are set to 0). This fit may however underestimate the magnitude of epistatic effects, because they are only used to explain that part of the variance, which cannot be explained by main effects.
- The HL_ε_ are derived from the HL by scaling the epistatic effects by a factor *ε* (i.e. by replacing the *ε_ij;kl_* with *ε_ij;kl_ ε*).

Figure S1 shows the predictive power of the different models.

### Environments

If not stated otherwise, the RC values underlying the fitness-landscapes RL, HL and HL_ε_ are measured in the absence of drugs. In addition we consider 15 alternative versions of the RL based on RC values measured in the presence of 15 different single drugs. The drugs used here are the protease inhibitors amprenavir (AMP), indinavir (IDV), lopinavir (LPV), nelfinavir (NFV), ritonavir (RTV), and saquinavir (SQV), the 6 nucleoside reverse transcriptase inhibitors abacavir (ABC), didanosine (ddI), lamivudine (3TC), stavudine (d4T), zidovudine (ZDV), and tenofovir (TFV) and the non-nucleoside reverse transcriptase inhibitors delavirdine (DLV), efavirenz (EFV), and nevirapine (NVP). For each drug, the replicative capacity of a virus on drugs was given by the interpolated value measured at the drug concentration at which the NL4-3 based control virus has 10% of its replicative capacity in the absence of drug (i.e. the IC90 for NL4-3 is used as the reference drug concentration for every subsequent measurement) [21].

### Characteristics of Fitness Landscapes

The landscapes are characterized by adaptive, neutral and random walks. Each walk consists of a series/succession of genotypes *s^0^→s^1^→s^2^→s^3^*…. . The different types of walks differ with respect to the updating rule (i.e. on how genotype *s^k+1^* is determined from *s^k^*). Unless stated otherwise the start genotype of each walk is chosen randomly, i.e. at each position one of the possible alleles is chosen randomly and independently from alleles at the other positions.

The ruggedness of the fitness landscapes is measured as the number of different end-points reached from a pre-specified number of steepest-ascent hill climbing walks (SAHCW) starting from different, random start genotypes. In each step *s^k^*→*s^k+1^* of a SAHCW, the fitness of all single mutants of *s^k^* is determined. If the single mutant with the maximal fitness (*s^max^*) is less fit than *s^k^*, the walk is terminated, as *s^k^* represents a local maximum. Otherwise, the fittest single mutant *s^max^* is chosen as the next genotype in the walk *(s^k+1^ = s^max^)*.

The neutrality of fitness landscapes is measured as the range explored by quasi-neutral walks (QNW) of a pre-specified length *L* (typically 1000 steps). In each step *s^k^*→*s^k+1^* of a QNW, a single random amino-acid substitution is performed on the genotype *s^k^*, yielding the genotype *s^k^'*. If the log-fitness of *s^k^'* differs by less than ε from the log-fitness of *s^k^*, then *s^k^'* is chosen as the next step in the QNW (*s^k+1^* = *s^k^'*). If the difference is larger than or equal to *ε*, the step is rejected and an alternative single mutant of *s^k^* is probed in the same way for its neutrality etc. If after 10^4^ trials, no quasi-neutral mutation has been found, the QNW stays at *s^k^* (*s^k+1^* = *s^k^*). The range of such a neutral walk is determined as the maximal Hamming distance of one the genotypes *s* ^1^….*s^L^* from the start-genotype *s*_0_.

The correlation length of a fitness landscape is measured as the inverse decay rate of the autocorrelation of the log-fitness along random walks (RW). Specifically, a pre-specified number (typically 10^5^) of random walks are initiated each from a different random start genotype. In each step of a given RW a single randomly chose amino acid substitution is performed. The autocorrelation after *k* steps is then determined as
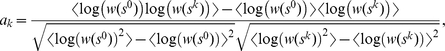
where the brackets refer to averaging over all random walks performed. An exponential decay is then fitted to these autocorrelation coefficients by performing a linear least-square fit according to
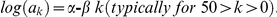
The correlation length is then given by 1/β.

## Supporting Information

Figure S1 Predictive power (measured as the fraction of the deviance explained) of the fitness models underlying the fitness landscapes considered. The dashed line corresponds to the RL. Points correspond to the HL_ε_ for different values of ε. See Hinkley at al. [21] for details on how predictive power was measured.
(PDF)

## References

[1] Wright S (1931). Evolution in Mendelian Populations.. Genetics.

[2] Collins JA, Thompson MG, Paintsil E, Ricketts M, Gedzior J (2004). Competitive Fitness of Nevirapine-Resistant Human Immunodeficiency Virus Type 1 Mutants.. J Virol.

[3] Deforche K, Camacho R, Van Laethem K, Lemey P, Rambaut A (2008). Estimation of an in vivo fitness landscape experienced by HIV-1 under drug selective pressure useful for prediction of drug resistance evolution during treatment.. Bioinformatics.

[4] Poelwijk FJ, Kiviet DJ, Weinreich DM, Tans SJ (2007). Empirical fitness landscapes reveal accessible evolutionary paths.. Nature.

[5] Weinreich DM, Delaney NF, DePristo MA, Hartl DL (2006). Darwinian Evolution Can Follow Only Very Few Mutational Paths to Fitter Proteins.. Science.

[6] Whitlock MC, Bourguet D (2000). Factors affecting the genetic load in Drosophila: synergistic epistasis and correlations among fitness components.. Evolution.

[7] Franke J, Klozer A, de Visser JA, Krug J Evolutionary accessibility of mutational pathways.. PLoS Comput Biol.

[8] O'Maille PE, Malone A, Dellas N, Andes Hess B, Smentek L (2008). Quantitative exploration of the catalytic landscape separating divergent plant sesquiterpene synthases.. Nat Chem Biol.

[9] Fontana W, Stadler PF, Bornberg-Bauer EG, Griesmacher T, Hofacker IL (1993). RNA folding and combinatory landscapes.. Phys Rev E Stat Phys Plasmas Fluids Relat Interdiscip Topics.

[10] Pitt JN, Ferre-DAmare AR Rapid Construction of Empirical RNA Fitness Landscapes.. Science.

[11] Lunzer M, Miller SP, Felsheim R, Dean AM (2005). The Biochemical Architecture of an Ancient Adaptive Landscape.. Science.

[12] Kauffman S, Levin S (1987). Towards a general theory of adaptive walks on rugged landscapes.. J Theor Biol.

[13] Weinberger E (1990). Correlated and uncorrelated fitness landscapes and how to tell the difference.. Biological Cybernetics.

[14] van Nimwegen E (2006). Epidemiology. Influenza escapes immunity along neutral networks.. Science.

[15] Fontana W, Schuster P (1998). Continuity in evolution: on the nature of transitions.. Science.

[16] Kimura M (1983).

[17] Koelle K, Cobey S, Grenfell B, Pascual M (2006). Epochal evolution shapes the phylodynamics of interpandemic influenza A (H3N2) in humans.. Science.

[18] Gavrilets S (2004).

[19] Bonhoeffer S, Chappey C, Parkin NT, Whitcomb JM, Petropoulos CJ (2004). Evidence for positive epistasis in HIV-1.. Science.

[20] Costanzo M, Baryshnikova A, Bellay J, Kim Y, Spear ED The genetic landscape of a cell.. Science.

[21] Hinkley T, Martins J, Chappey C, Haddad M, Stawiski E (2011). A systems analysis of mutational effects in HIV-1 protease and reverse transcriptase.. Nat Genet.

[22] Johnson VA, Brun-Vezinet F, Clotet B, Gunthard HF, Kuritzkes DR (2010). Update of the drug resistance mutations in HIV-1: December 2010.. Top HIV Med.

[23] Martinez-Cajas JL, Wainberg MA (2007). Protease inhibitor resistance in HIV-infected patients: molecular and clinical perspectives.. Antiviral Res.

[24] Doyon L, Tremblay S, Bourgon L, Wardrop E, Cordingley MG (2005). Selection and characterization of HIV-1 showing reduced susceptibility to the non-peptidic protease inhibitor tipranavir.. Antiviral Res.

[25] Otto SP, Barton NH (2001). Selection for recombination in small populations.. Evolution.

[26] Keightley PD, Otto SP (2006). Interference among deleterious mutations favours sex and recombination in finite populations.. Nature.

[27] Fontana W, Konings DAM, Stadler PF, Schuster P (1993). Statistics of Rna Secondary Structures.. Biopolymers.

[28] Kouyos RD, von Wyl V, Hinkley T, Petropoulos CJ, Haddad M (2011). Assessing predicted HIV-1 replicative capacity in a clinical setting.. PLoS Pathog.

[29] Petropoulos CJ, Parkin NT, Limoli KL, Lie YS, Wrin T (2000). A novel phenotypic drug susceptibility assay for human immunodeficiency virus type 1.. Antimicrob Agents Chemother.

[30] Mayer JE, Montroll E (1941). Molecular distribution.. Journal of Chemical Physics.

