# Supplementary figures and images for: Exploring the Complexity of the HIV-1 Fitness Landscape
Source: PLoS Genet. 2012 Mar 8;8(3):e1002551. doi: 10.1371/journal.pgen.1002551 (PMC3297571; doi:10.1371/journal.pgen.1002551)

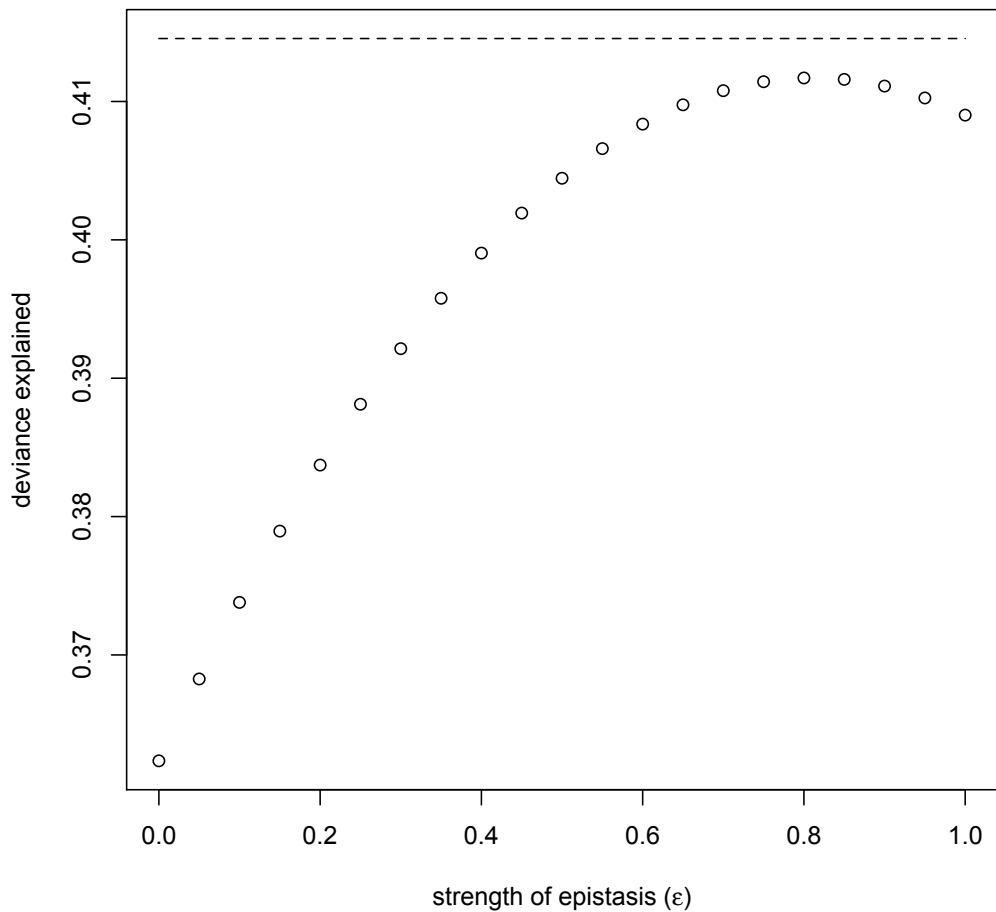

Supplement: Figure S1 — Predictive power (measured as the fraction of the deviance explained) of the fitness models underlying the fitness landscapes considered. The dashed line corresponds to the RL. Points correspond to the HLε for different values of ε. See Hinkley at al. [21] for details on how predictive power was measured. (PDF) [file pgen.1002551.s001.pdf]
